# Increased risk of type 2 diabetes after traumatic amputation: a nationwide retrospective cohort study

**DOI:** 10.3389/fendo.2024.1437860

**Published:** 2025-01-07

**Authors:** Jung Eun Yoo, Dagyeong Lee, Bongseong Kim, Won Hyuk Chang, Sang-Man Jin, Kyungdo Han, Dong Wook Shin

**Affiliations:** ^1^ Department of Family Medicine, Healthcare System Gangnam Center, Seoul National University Hospital, Seoul, Republic of Korea; ^2^ Department of Family Medicine, Seoul National University College of Medicine, Seoul, Republic of Korea; ^3^ Department of Family Medicine, Hallym University Dongtan Sacred Heart Hospital, Hwaseong, Republic of Korea; ^4^ Department of Statistics and Actuarial Science, Soongsil University, Seoul, Republic of Korea; ^5^ Department of Physical and Rehabilitation Medicine, Center for Prevention and Rehabilitation, Heart Vascular Stroke Institute, Samsung Medical Center Sungkyunkwan University School of Medicine, Seoul, Republic of Korea; ^6^ Division of Endocrinology, Department of Internal Medicine, Samsung Medical Center, Sungkyunkwan University School of Medicine, Seoul, Republic of Korea; ^7^ Department of Family Medicine/Supportive Care Center, Samsung Medical Center, Sungkyunkwan University School of Medicine, Seoul, Republic of Korea; ^8^ Department of Clinical Research Design & Evaluation, Samsung Advanced Institute for Health Science & Technology (SAIHST), Sungkyunkwan University, Seoul, Republic of Korea

**Keywords:** amputation, trauma, type 2 diabetes, disability, proximal amputation

## Abstract

**Background:**

Amputation confers disabilities upon patients and is linked to cardiometabolic morbidity and mortality. We aimed to compare the incidence of type 2 diabetes (T2DM) between individuals following amputation with those of the general population.

**Methods:**

We performed a population-based retrospective cohort study using the Nationwide Health Insurance Service database. A total of 21,343 individuals with amputation during 2010–2018 and their 1:3 age- and sex-matched controls was included. We conducted Cox proportional hazard analysis to calculate the risk of T2DM among individuals with amputation.

**Results:**

During the 4.2 ± 2.5 year mean follow-up period, there were 912 incident T2DM cases (10.7 per 1,000 person-years) among individuals with amputation. Individuals with amputation had a higher risk for T2DM (adjusted hazard ratio [aHR] 1.11, 95% confidence interval [CI] 1.03–1.20) compared with matched controls. The risks were increased further when accompanied with disability; those with severe disability had a higher risk of T2DM (aHR 1.77, 95% CI 1.20–2.60) than matched controls. Individuals with proximal upper limb amputation (aHR 1.10, 95% CI 1.02–1.18) and proximal lower limb amputation (aHR 3.60, 95% CI 1.50–8.64) had a higher risk of T2DM compared with matched controls.

**Conclusions:**

Individuals with amputation were at significantly greater risk for T2DM than the general population, particularly those with severe disability and proximal amputation. Innovative strategies that improve and support the long-term T2DM risk for severely injured individuals with proximal amputation are warranted.

## 1 Introduction

Amputation causes disability, severely impairs patient mobility and physical function, and has imposed a significant global health burden (1). Individuals with amputation represent a population with wide-ranging medical needs related not only to the amputation itself, but also to long-term secondary complications (2). Particularly, individuals with trauma-related amputations typically sustain their injuries at relatively young age and have a longer post-amputation life than those undergoing amputations later in life (2), emphasizing the need for extensive long-term health care and rehabilitation needs.

The prevalence of type 2 diabetes (T2DM) is increasing globally, with increasing numbers diagnosed at younger ages (3). T2DM and its associated complications affect individuals’ functional capacities and quality of life, leading to significant morbidity and premature mortality (4). Among noncommunicable disease, T2DM is the only significant disease for which the risk of premature death is increasing rather than decreasing (5). Amputation negatively impacts behavioral and social factors related to obesity, nutrition, and physical activity, which can contribute to T2DM.

Interestingly, even when amputation occurs as the result of an accident or injury, individuals with amputation appear to be at higher risk of cardiometabolic risk factors (6–10) compared to the general population. However, most studies have focused on glucose intolerance defined as fasting glucose level ≥100 mg/dL (6, 7, 10), insulin levels (11, 12), or metabolic syndrome (9) rather than actual diagnosis of T2DM, resulting in lack of data about how T2DM risk changes after amputation. Research also is limited by difficulties in generalizing findings due to relatively small populations [the largest study has only 588 individuals with amputation (9)], cross-sectional study design (6–8, 10–12), or old data [collected before 2000 (6, 8, 11, 12)].

To understand better the association of amputation with the risk of T2DM, large-scale, nationally representative data from the Korean National Health Insurance Service (NHIS) were analyzed. We compared the risk of incident T2DM between individuals’ post-amputation and the general population. We also investigated how the severity of disability from amputation and the levels of amputation affect the association with T2DM.

## 2 Methods

### 2.1 Patient consent

Anonymized and de-identified information was used for analyses; therefore, informed consent was not required.

### 2.2 Data source

In Korea, all residents are assigned unique personal identification numbers that enable the National Health Insurance Database (NHID) to link with various national databases. The NHID consists of five components: the eligibility database, which contains income-related insurance contributions and demographic information; the national health screening database, which collects health behavior data and bio-clinical variables; the health care utilization database, which documents inpatient and outpatient services; the long-term care insurance database, which contains information on activities of daily living; and the health care provider database, which contains details of health care facilities and resources. This structure allows comprehensive tracking of health outcomes over time by linking routine health screening results with medical claims, providing a robust dataset for analysis (13).

The NHIS is the single mandatory health insurance in Korea and covers approximately 97% of the Korean population; the remaining 3% with the lowest income are covered by the Medical Aid Program. NHIS manages all administrative processes and reimburses medical providers and pharmacies for claims of medical and pharmacy services. The NHIS also provides biennial health screening for all Koreans aged 40 and above. The health screenings consist of anthropometric measurements (height, weight, blood pressure, etc.), health behaviors (smoking, alcohol consumption, physical activity, etc.), and laboratory results (lipid profiles, blood glucose, etc.) (14). This database serves as a valuable and extensive resource for both clinical and public health research (15).

### 2.3 Study population

A total of 59,392 individuals who underwent traumatic amputation between January 1, 2010, and December 31, 2018, was identified. The criteria for amputation were determined based on the International Classification of Diseases, 10th revision, including codes Z89 (acquired absence of limb, site unspecified), S48 (traumatic amputation of shoulder joint), S58 (traumatic amputation of forearm), S68 (traumatic amputation of wrist, finger), S78 (traumatic amputation of hip), S88 (traumatic amputation of knee), and S98 (traumatic amputation of ankle, toe). The study included individuals aged 20 years or older who had undergone health screening within 2 years before the index date, totaling 27,810 individuals.

Individuals were excluded based on ICD-10 codes: 1) those diagnosed with diabetes with unspecified complications such as lower limb ulcer (e.g., diabetic foot) (ICD-10 codes: E10.6, E11.6, E12.6, E13.6, E14.6, L97, and E11.621) (n = 500); this did not exclude individuals with clearly specified complications such as diabetic retinopathy or nephropathy; and 2) individuals with thromboangiitis obliterans (I73.1) (n = 140) or arterial embolism and thrombosis (I74) (n = 1002). Exclusions also applied to those with T2DM based on ICD-10 code (E11–14) before amputation (n = 2,839) or with fasting glucose level ≥126 mg/dL during health screening upon enrollment (n = 787). Participants with missing information were excluded (n = 926). To mitigate reverse causality, individuals with less than 1 year of follow-up were excluded (n = 273). Ultimately, the study included 21,343 individuals with amputation.

**Figure 1:**
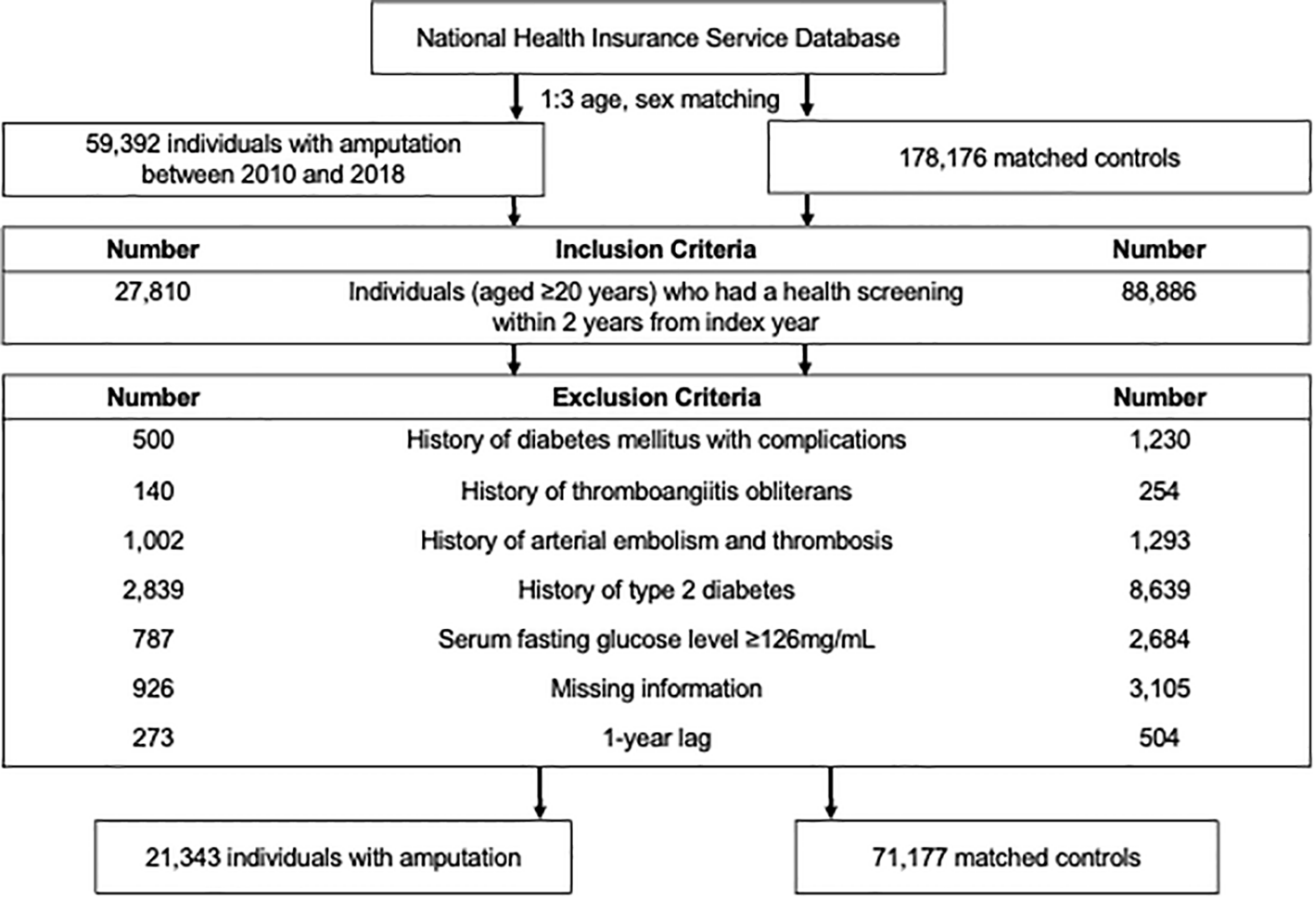
Flow chart of the study population.

We selected the amputation-free controls matched by age and sex to assess the impact of amputation on the subsequent development of T2DM. For the control group, 1:3 age and sex matching were conducted, assigning matched controls an index date corresponding to the amputation date for individuals with amputation. The same exclusion criteria were applied to control subjects. The study population selection process is depicted in **Figure 1**.

### 2.4 Disability and levels of amputation

We utilized data from the Korean National Disability Registration System (KNDRS) to determine post-amputation disability status and severity (16). In Korea, individuals with disabilities register to access government-provided social benefits. Specialized physicians document disability diagnoses through physical examinations and X-rays, requiring the submission of validated documents. The disability degree ranges from grade 1, the most severe, to grade 6, the mildest. Generally, amputations at proximal sites result in more severe disability. For instance, grade 1 disability from upper extremity amputation involves amputation above the wrist joint for both arms, while grade 6 disability involves amputation of one thumb above the interphalangeal joint (**Supplementary Tables 1**, **2**). In this study, individuals registered in KNDRS within 1 year from the index date were classified into the disability group and then further categorized as mild to moderate (grades 4-6) and severe (grades 1–3) disability groups. Conversely, those not registered in KNDRS were classified into the no disability group (e.g., amputation of one finger below the PIP joint).

To explore the association between amputation levels and T2DM, additional analyses were conducted, considering the ICD-10 codes indicating amputation levels. Upper limb amputations were grouped into shoulder/upper arm, forearm, and wrist/hand. Lower limb amputations were divided into hip/thigh, lower leg, and ankle/foot.

### 2.5 Study outcomes and follow-Up

The main outcome of this study was newly diagnosed T2DM. A T2DM diagnosis was confirmed when the fasting glucose level was ≥126 mg/dL or when there were claims associated with the ICD-10 code E11–14 with a prescription for antidiabetic medications. The study population was observed from 1 year after the index date (lag period) until the onset of incident T2DM, death, the censor date, or the end of the study period (December 31, 2019), whichever came first.

### 2.6 Covariates

Baseline information for individuals with amputation and the matched control group was obtained from disease codes in the year prior to amputation/index date and health screening results within 2 years before the amputation/index date. We collected sociodemographic information, including age, gender, income level, and residential area, from the NHIS database. Regarding alcohol consumption, individuals were categorized into non-drinker, mild to moderate drinker (consuming <30 g of alcohol per day), and heavy drinker (≥30 g/day). Smoking habits were classified into never-smoker, former smoker, and current smoker. Regular physical activity was defined as engaging in moderate physical activity for over 30 minutes per session at least 5 days per week or vigorous physical activity for more than 20 minutes per session at least 3 days per week.

Comorbidities were identified by analyzing both claims data preceding the screening date and the outcomes of health screenings. Hypertension was diagnosed if there was a claim with I10–I13 or I15 and evidence of antihypertensive medication use or systolic/diastolic blood pressure ≥140/90 mmHg. Dyslipidemia was characterized by a claim with E78 codes and the use of lipid-lowering medications or a total cholesterol level ≥240 mg/dL.

### 2.7 Statistical analysis

A descriptive analysis of the study subjects was performed to compare the baseline characteristics of individuals with amputation and their matched controls. Cox proportional hazards regression analysis was performed to calculate the hazard ratio (HR) and 95% confidence interval (CI). The HR was obtained in two steps: model 1, which was unadjusted, and model 2, which was adjusted for variables of age, sex, socioeconomic status (income level and place of residence), health behaviors (smoking, alcohol consumption, and physical activity), body mass index, fasting glucose level, and the presence of comorbidities (hypertension and dyslipidemia).

Statistical analyses were conducted using SAS version 9.4 (SAS Institute Inc., Cary, NC, USA), and a P-value < 0.05 was considered statistically significant.

## 3 Results

### 3.1 Baseline characteristics

The baseline characteristics of the study population are presented in **Table 1**. Among 21,343 individuals with amputation, the mean age at baseline was 53.3 ± 12.2 years. Compared with controls, individuals with amputation were more likely to have lower income, be heavy drinkers and/or current smokers, and be less likely to engage in physical activity. They had higher prevalence of hypertension and dyslipidemia than matched controls (P < 0.05 for all). Individuals with amputation and disability included larger proportions of low-income patients and heavy alcohol drinkers. Amputees also exhibited a higher prevalence of comorbidities such as hypertension and dyslipidemia than those without disability (P < 0.001 for all).

**Table 1:** Baseline characteristics of the study population.

	Study population			Individuals with amputation		
	Matched controls (*N* = 71,177)	Individuals with amputation (*N* = 21,343)	*P*-value	Without disability (*N* = 19,652)	With disability (*N* = 1,691)	*P*-value
Age, years	52.5 ± 11.9	53.3 ± 12.2	< 0.001	52.8 ± 12.2	59.1 ± 11.0	< 0.001
Sex, male, No. (%)	55,144 (77.5)	16,104 (75.5)	< 0.001	14,731 (75.0)	1,373 (81.2)	< 0.001
Income of lowest 20%, No. (%)	12,037 (16.9)	3,991 (18.7)	< 0.001	3,637 (18.5)	354 (20.9)	< 0.001
Urban residency, No. (%)	32,535 (45.7)	7,138 (33.4)	< 0.001	6,638 (33.8)	500 (29.6)	< 0.001
Alcohol consumption			< 0.001			< 0.001
None	30,978 (43.5)	9,662 (45.3)		8,828 (44.9)	834 (49.3)	
Mild to moderate	33,037 (46.4)	8,984 (42.1)		8,354 (42.5)	630 (37.3)	
Heavy	7,162 (10.1)	2,697 (12.6)		2,470 (12.6)	227 (13.4)	
Smoking status			< 0.001			< 0.001
Never	32,095 (45.1)	9,684 (45.4)		8,943 (45.5)	741 (43.8)	
Former	17,510 (24.6)	4,473 (21.0)		4,076 (20.7)	397 (23.5)	
Current	21,572 (30.3)	7,186 (33.7)		6,633 (33.8)	553 (32.7)	
Regular physical activity, No. (%)	15,515 (21.8)	3,742 (17.5)		3,460 (17.6)	282 (16.7)	
Anthropometrics						
Body mass index, kg/m^2^	24.1 ± 3.1	24.0 ± 3.1	< 0.001	24.0 ± 3.1	24.0 ± 3.1	< 0.001
Waist circumference, cm	82.4 ± 8.4	81.9 ± 8.4	< 0.001	81.8 ± 8.4	83.2 ± 8.2	< 0.001
Systolic blood pressure, mmHg	123.6 ± 14.2	123.6 ± 14.5	0.595	123.5 ± 14.5	125.6 ± 15.0	< 0.001
Diastolic blood pressure, mmHg	77.3 ± 9.8	77.1 ± 9.9	0.091	77.1 ± 9.9	77.9 ± 10.1	< 0.001
Laboratory findings						
Fasting glucose, mg/dL	95.1 ± 11.2	94.9 ± 11.5	0.027	94.8 ± 11.5	96.0 ± 11.7	< 0.001
Total cholesterol, mg/dL	198.0 ± 36.4	196.3 ± 36.8	< 0.001	196.4 ± 36.7	195.6 ± 37.5	< 0.001
Comorbidity						
Hypertension, No. (%)	21,172 (29.8)	6,514 (30.5)	0.030	5,849 (29.8)	665 (39.3)	< 0.001
Dyslipidemia, No. (%)	16,406 (23.1)	4,723 (22.1)	0.005	4,286 (21.8)	437 (25.8)	< 0.001

Data are expressed as mean ± standard deviation or number (%).

### 3.2 Risk of subsequent T2DM among people with amputation

**Figure 2:**
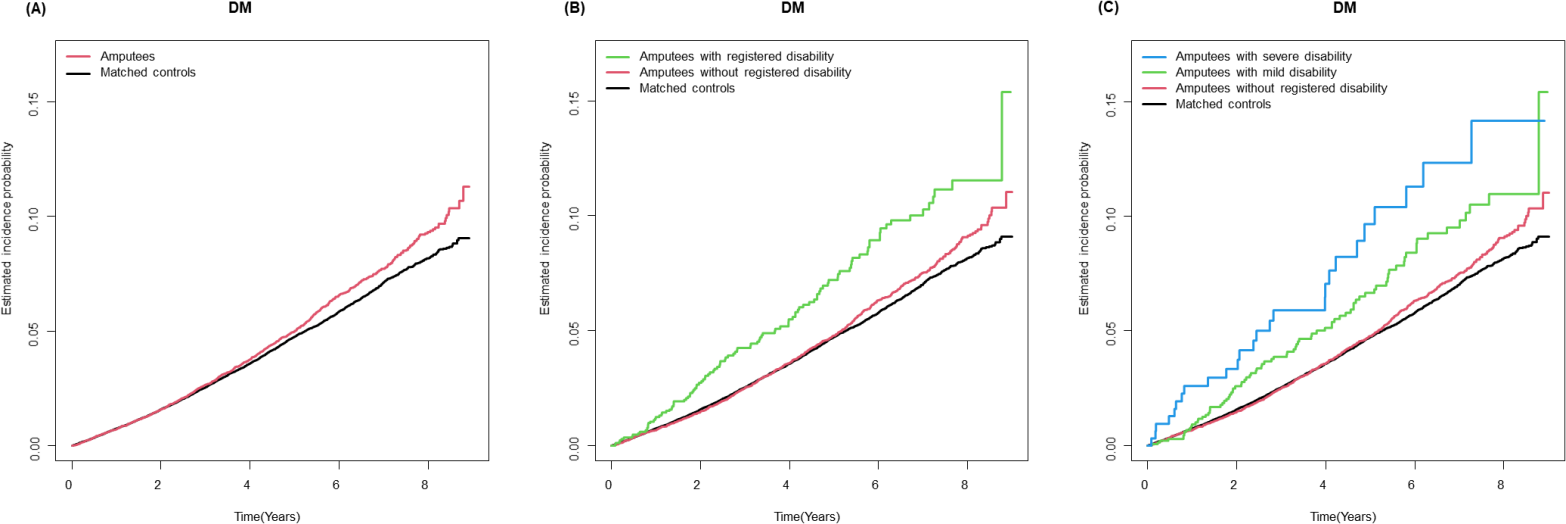
Kaplan-Meier curves for incidence of type 2 diabetes in individuals with amputation compared with matched controls. **(A)** Amputation status **(B)** Amputation status and registered disability **(C)** Amputation status and severity of registered disability.

During a mean follow-up period of 4.2 ± 2.5 years after a 1-year lag period, 912 incident T2DM cases (10.7 per 1,000 person-years) were observed among individuals with amputation (**Table 2**). Kaplan–Meier curves shows that the incidence probabilities of T2DM in individuals with amputation were higher than controls (log-rank P < 0.001) (**Figure 2**). Compared with matched controls, people with amputation had a higher risk of T2DM (adjusted HR [aHR] 1.11 95% CI 1.03–1.20). The risks further increased with the presence of disability (aHR 1.27, 95% CI 1.05–1.54). Furthermore, those with severe disability had the highest risk of T2DM (aHR 1.77, 95% CI 1.20–2.60). By level of amputation, individuals with wrist/hand amputation (aHR 1.10, 95% CI 1.02–1.18) and hip/thigh amputation (aHR 3.60, 95% CI 1.50–8.64) had a higher risk of T2DM compared with matched controls.

**Table 2:** Hazard ratios and 95% confidence intervals for the incidence of diabetes mellitus among individuals with amputation compared to the matched controls.

	Number	Event number (n)	IR per 1,000 person-years	Model 1 HR (95% CI)	Model 2 aHR (95% CI)
Matched controls	71,177	3,022	9.8	1 (ref.)	1 (ref.)
Individuals with amputation	21,343	971	10.7	1.10 (1.02, 1.18)	1.11 (1.03, 1.20)
By presence of registered disability					
No disability	19,652	859	10.3	1.06 (0.98, 1.14)	1.09 (1.01, 1.18)
Disability	1,691	112	15.1	1.53 (1.27, 1.85)	1.27 (1.05, 1.54)
By severity of disability					
Mild (grade 4-6)	1,376	86	14.1	1.43 (1.15, 1.77)	1.17 (0.95, 1.46)
Severe (grade 1-3)	315	26	19.5	2.00 (1.36, 2.94)	1.77 (1.20, 2.60)
By levels of amputation					
Acquired absence of limb (site unspecified)	38	2	24.4	2.86 (0.71, 11.43)	1.71 (0.43, 6.86)
Shoulder/Upper arm	58	2	8.2	0.84 (0.21, 3.36)	1.06 (0.27, 4.24)
Forearm	87	4	9.7	0.97 (0.37, 2.59)	0.98 (0.37, 2.62)
Wrist/Hand	20,550	921	10.6	1.08 (1.01, 1.17)	1.10 (1.02, 1.18)
Hip/Thigh	31	5	44.9	4.80 (2.00, 11.54)	3.60 (1.50, 8.64)
Lower leg	119	9	17.2	1.75 (0.91, 3.37)	1.57 (0.82, 3.02)
Ankle/Foot	460	28	14.5	1.48 (1.02, 2.15)	1.39 (0.96, 2.02)

IR, incidence rate; HR, hazard ratio; CI, confidence interval.

Model 1: Crude model.

Model 2: Adjusted for age, sex + socioeconomic position (income level and place of residence), smoking, alcohol consumption, physical activity, body mass index, fasting glucose level, hypertension, and dyslipidemia.

## 4 Discussion

The present study investigated the risk of T2DM among individuals with amputation compared to matched controls and showed an 11% higher risk of T2DM after adjusting for potential confounders. The association was robust among those with severe disability and proximal lower limb amputation. The major strengths of our study include the use of national claims data, which enabled a larger sample size, as well as inclusion of age- and sex-matched controls, with low attrition rates. Moreover, adjusting for various possible confounders was possible through linkages with health screening data.

We observed an increased T2DM risk among individuals with amputation compared with matched controls. Following amputation, individuals are predisposed to numerous unfavorable changes in health. These include changes in body composition characterized by muscle atrophy in the amputated limb due to decreased use of the muscle tissue as well as reduced muscle fiber size and the development of central and peripheral adiposity (17). Possible explanations for the notable increase in fat mass may be related to lifestyle changes such as unbalanced diet, increased alcohol intake and smoking, or decreased physical activity from before the injury (17–19). We observed that the risk of T2DM increased with the severity of disability after amputation, highlighting the impact of decreased physical activity after amputation on the development of T2DM. Physical inactivity is associated with decreased energy expenditure that results in a positive energy balance, leading to obesity, insulin resistance, and T2DM. Moreover, individuals with amputation have increased sympathetic nerve activity, leading to greater blood glucose fluctuations and insulin resistance (12). Hyperinsulinemia causes excessive stimulation of the carotid body, leading to increased sympathetic adrenal activity and blood flow, forming a vicious circle and exacerbating abnormal glucose metabolism (20).

In addition, those with amputation, particularly, with disability, are more likely to secondary conditions following amputation, such as depression (21) or chronic pain (22), which contribute to the development of T2DM.

When considering levels of amputation, an increased risk of T2DM was prominent in proximal lower limb amputations. Individuals with amputation at the hip/thigh level had a greater than 3-fold increase in T2DM risk compared with matched controls. From a physiological perspective, contractile activity of slow oxidative muscle, most notably the soleus and vastus intermedius muscles, has been reported to be effective at improving metabolic and glucose regulation (23). Furthermore, previous studies have reported that the level of amputation is a significant factor in the severity of muscle atrophy (24, 25) and weight gain (18). For example, 2 years after amputation, people with a more proximal amputation had an 8–9% weight gain on average, whereas people with a more distal amputation had a 3–6% weight gain (18). Barriers related to prosthesis fitting are likely more limiting for individuals with proximal lower limb amputation than distal lower limb amputation and may explain the differential weight gain by amputation level.

The clinical implication of our study is that amputation has a longitudinal impact on many areas outside of the residual limb itself, and individuals with amputation are at significantly higher risk for incident T2DM. Even when patients underwent amputation due to trauma, they are at higher risk for T2DM. A previous study demonstrated that the age-standardized prevalence consistently increased with a more rapid risk among people with disabilities compared to people without disabilities (26). As current guidelines recommend screening for T2DM in individuals with risk factors such as overweight or obese and sedentary lifestyle (27), our data support that individuals with amputation should be candidates for early/frequent T2DM screening. These conditions also highlight the importance of early behavior intervention including proper physical activity through rehabilitation and nutritional education (28).

There were several limitations to our study. First, we defined the incident T2DM based on claims data, which may be influenced by healthcare accessibility. For example, the risk of T2DM may have been underestimated due to the limited access to preventive care for individuals with amputations. Second, data on oral glucose tolerance tests and glycosylated hemoglobin assays were unavailable from the NHIS database, possibly leading to an underestimation of the incidence of T2DM. Third, a limitation of this study was the inability to analyze changes in body weight and physical activities before and after amputation, as the dataset used in this study does not include records of post-amputation health examinations. As a result, we were unable to discriminate how changes in body weight and physical activities influence the development of T2DM after amputation. Forth, this was a retrospective study, and the findings should be interpreted accordingly. We applied a 1-year lag period following amputation to minimize the possible effects of reverse causality. Last, the generalizability of the results may be limited by the ethnic homogeneity of the database.

## 5 Conclusion

In conclusion, our findings indicate that individuals with amputation are at an increased risk of developing T2DM compared to matched controls. This risk is particularly pronounced among those with severe disability and proximal lower limb amputation. This highlights the crucial importance of implementing proactive public health strategies to address the specific needs of individuals who have undergone amputation. Such strategies must ensure that appropriate intervention and support are provided at the earliest opportunity in order to minimize the impact of amputation on overall health outcomes.

## Data Availability

Publicly available datasets were analyzed in this study. This data can be found here: The Korean National Health Insurance Service/The database is open to all researchers whose study protocols are approved by the official review committee.
