# Supplementary material for: Increased risk of type 2 diabetes after traumatic amputation: a nationwide retrospective cohort study
Source: Front Endocrinol (Lausanne). 2025 Jan 7;15:1437860. doi: 10.3389/fendo.2024.1437860 (PMC11746081; doi:10.3389/fendo.2024.1437860)
Supplement: Supplementary file 1 [file DataSheet1.docx]

**Supplementary Table S1.** Definitions of severity degree in upper extremity amputation

| Grade | Definitions |
| --- | --- |
| 1 | Amputation above the wrist joint of both arms |
| 2 | Amputation of both thumbs above the IP joint and all 2^nd^ to 5^th^ fingers above the PIP joint  Amputation above the elbow joint of one arm |
| 3 | Amputation of both thumbs above the IP joint and the 2^nd^ finger above the PIP joint  Amputation of one thumb above the IP joint and all 2^nd^ to 5^th^ fingers above the PIP joint |
| 4 | Amputation of both thumbs above the IP joint  Amputation of one thumb above the IP joint and the 2^nd^ finger above the PIP joint  Amputation of one thumb above the IP joint and another two fingers above the PIP joint |
| 5 | Amputation of one thumb above the IP joint and another finger above the PIP joint  Amputation of one thumb above the MCP joint  Amputation of three fingers including the 2^nd^ finger above the PIP joint |
| 6 | Amputation of one thumb above the IP joint  Amputation of two fingers including the 2^nd^ finger above the PIP joint  Amputation of all 3^rd^ to 5^th^ fingers of one hand above the PIP joint |

IP, Interphalangeal; PIP, proximal interphalangeal; MCP, metacarpophalangeal

**Supplementary Table S2.** Definitions of severity degree in lower extremity amputation

| Grade | Definitions |
| --- | --- |
| 1 | Amputation of both legs above the knee joint |
| 2 | Amputation of both legs above the ankle joint |
| 3 | Amputation of both legs above the transverse tarsal joint (Chopart joint)  Amputation of one leg above the knee joint |
| 4 | Amputation of both legs above the tarsometatarsal joint (Lisfranc joint)  Amputation of one leg above the ankle joint |
| 5 | Amputation of both big toes above the interphalangeal joint and the 2^nd^ to 5^th^ toes above the proximal interphalangeal joint  Amputation of one leg above the transverse tarsal joint (Chopart joint) |
| 6 | Amputation of one leg above the tarsometatarsal joint (Lisfranc joint) |
